# Enhancing lithic analysis: Introducing *3D-EdgeAngle* as a semi-automated 3D digital method to systematically quantify stone tool edge angle and design

**DOI:** 10.1371/journal.pone.0295081

**Published:** 2023-11-30

**Authors:** Lisa Schunk, Anja Cramer, Konstantin Bob, Ivan Calandra, Guido Heinz, Olaf Jöris, João Marreiros

**Affiliations:** 1 Faculty of Historical and Pedagogical Sciences, Institute of Archaeology, University of Wroclaw, Wrocław, Poland; 2 TraCEr, Laboratory for Traceology and Controlled Experiments, MONREPOS Archaeological Research Centre and Museum for Human Behavioural Evolution, LEIZA, Neuwied, Germany; 3 Department of Prehistoric and Protohistoric Archaeology, Institute of Ancient Studies, Johannes Gutenberg University, Mainz, Germany; 4 LEIZA, Leibniz Zentrum für Archäologie, Mainz, Germany; 5 Scientific Computing and Bioinformatics, Institute of Computer Science, Johannes Gutenberg University, Mainz, Germany; 6 IMPALA, Imaging Platform at LEIZA, LEIZA, Neuwied, Germany; 7 MONREPOS Archaeological Research Centre and Museum for Human Behavioural Evolution, LEIZA, Schloss Monrepos, Neuwied, Germany; 8 ICArEHB, Interdisciplinary Center for Archaeology and Evolution of Human Behaviour, University of Algarve, Faro, Portugal; Sapienza University of Rome: Universita degli Studi di Roma La Sapienza, ITALY

## Abstract

In stone tool studies, the analysis of different technological and typological features is known to provide distinct but interrelated information on the design and use of artefacts. The selection of these features can potentially influence the understanding and reconstruction of past human technological behaviour across time. One feature frequently part of a standard lithic analysis is the measurement of edge angles. The angle of an edge, unmodified or shaped by retouch and an integral part of the overall tool design, is certainly a parameter that influences the interpretation of an artefact. The acuteness of an edge angle is often linked to aspects such as cutting, carving, or scraping efficiency and durability and thus, tool performance. Knowing the actual edge angle of a stone tool can therefore have important implications for its interpretation. In the case of edge angle analyses, manual measuring techniques have been established for many years in lithic studies. Here, we introduce a new method for accurate and precise edge angle measurements based on 3D data (hereafter *3D-EdgeAngle*). *3D-EdgeAngle* consists of a script-based, semi-automated edge angle measuring method applicable to 3D models. Unlike other methods, *3D-EdgeAngle* illustrates an objective way of measuring the edge angle at cross sections along the entire tool edge in defined steps and, moreover, allows measurements at different distances perpendicular to the edge by controlling three involved parameters. Thus, with this method, the edge angle can be measured at any point in a high resolution and scale of analysis. Compared to measurements taken manually, with this method random and systematic errors can be reduced significantly. Additionally, all data are reproducible and statistically evaluable. We introduce *3D-EdgeAngle* as a standard method to calculate edge angles with a highly accurate and systematic approach. With this method, we aim to improve the process of studying lithics and thus to increase the understanding of past human tool design.

## 1. Introduction

As being almost always preserved, stone artefacts represent one of the most crucial sources of information in the Stone Age archaeological record, serving as a window into the evolution of human technological and ecological adaptations. Interpretations of these tools are commonly based on technological, typological, and/or functional analyses. Each of these can hold a great potential for understanding and reconstructing past human behaviours. In the process of studying stone tools, varying technological and typological features are usually recorded to gain information not only about an artefact *per se*, but also about the relevance of certain technological and/or functional aspects in human decision-making processes underlaying tool design. Thus, improving the way archaeologists read the variability seen in the archaeological record is highly important. While aiming at increasing and enhancing the body of evidence from these features, the researcher also must select the most appropriate methods for characterisation and measurements depending on the research question.

In general, studying lithics always implies gaining information from several features illustrating aspects of tool design. Measuring certain attributes of lithics as for instance length, width and thickness allows quantifying tool design and offers the possibility of intra- and inter- assemblage comparisons [1–4]. Design includes a multitude of facets and goes beyond tool shape and morphology. It involves the workability of a raw material as a prerequisite and the human anticipation, interpretation, and execution of such workability. In particular, tool design also involves the engagement of the individual, who produced it, with the function of the tool and its interaction with the user [e.g. 5–8]. One integral part of the overall tool design is the tool’s active edge [9]. It can be described in a qualitative way (overall shape, profile, and intentional modification) or quantified by for example calculating edge curvature [10–12], edge sharpness, and the edge angle. Quantification of these features is not as straightforward as it might seem.

In the case of lithics, the edge angle describes the angle between the intersection of two surfaces, either the dorsal and ventral faces of a flaked blank or the two faces of a bifacial tool. Edge angles are strongly influenced by raw material shape and quality, technologies applied, tool use as well as by individual knapping skills, especially the ability to maintain acute angles during the process of reduction. In this regard, it is noteworthy that edge angles are not necessarily meant to be acute, as edge stabilisation and/or backing are functionally alternative edge modification strategies. The acuteness of the edge angle, however, often plays a role in the interpretation of the tool’s function. Highly correlated to the edge angle is the edge sharpness [13]. Both are distinct features, which need to be evaluated and measured differently. While lithic sharpness is more a parameter reflecting a snapshot in time due to its variability throughout use, the edge angle has a more significant and consistent influence on tool design.

Thus, the edge design of a tool is linked to aspects such as tool performance, durability, and efficiency [14–17]. Different ranges of edge angle values are often considered to reflect or to be optimised for the performance of a certain task and may explain to a certain degree technological and functional variability. This point can easily be underlined by a comparison with, for instance, modern cutting edges. Modern cutting tools are highly task-specifically designed, ranging from low edge angle values with less than 20° (scalpel, razor blades), medium values up to 40° (Japanese knives, standard cooking knives) to higher edge angles around 50° or 60° (hatchets, axes). Applied to the archaeological research, understanding edge design therefore presumes knowing the edge angle values. Simultaneously, it implies the identification and characterisation of the active part(s) of the edge. Furthermore, studies also point out the relation between use-wear traces and the edge angle [18,19], highlighting the importance of this specific feature.

Several techniques have been developed for measuring edge angles in lithic studies. Common manual techniques make use of the calliper, the goniometer [20] or the moulding method [21], to name only a few. The *‘caliper method’* calculates edge angle values from a thickness measurement taken by a customised calliper at a known distance from the edge of the artefact [20]. Following this method, the edge angle is measured at three locations (proximal, medial, and distal) on the edge. The arithmetic mean of the three values will give the result for the final edge angle value. The use of a goniometer also offers a manual possibility to measure the edge angle and is commonly used in archaeological studies. In that case, the artefact is placed with one surface against a bar of the goniometer and the value can be read directly from the goniometer scale. While these methods are prone to errors and lead to non-reproducible results, their goal is more to get an overall value per artefact. A more reliable, but time-consuming method is the moulding technique, which takes the different values along the entire edge into account. By taking moulds of the edge, it is possible to cut the mould at certain intervals based on the number of measurements needed. The information about the angle can be obtained via digital imaging analysis of the moulds without risking damaging the tool [21].

Besides these manual techniques, recently researchers have been focusing on the development of more advanced methods using 3D digital models [10,11,22–28]. The use of computer-based techniques combined with 3D models allows for the automated computation of edge angles from PCA (Principal Component Analysis) orientated artefacts [23,24,27–29]. Another possibility has been demonstrated by Valetta et al. [26]. Based on a 3D model of an artefact, the edge angle can be calculated by choosing an area representing the intersection between two surfaces. The method results in a mean angle value taking into account the error related to surface irregularities. Most recently, Macdonald et al. [11] demonstrated a method in which the edge curvature and edge angles get mathematically calculated using a multiscalar length-scale analysis. The analysis, however, is based on time-consuming and expensive microCT data.

As a recent development, the integration of algorithm-, script-based and automated methods considerably improves the possibilities to gain information, and leads to reproducible results as well as decreasing the random error. Nevertheless, all these methods result in an overall value per artefact, disregarding possible variability along the edge and overlooking certain details of a tool’s design, such as the effect of a retouch on the edge angle. To address and understand tool design, including all related aspects such as function, use, and tool (re-)sharpening, a mean value of the edge angle is not sufficient. With more detail, it is possible to address technological choices as well as the implications retouch and reduction have on the tool during its use-life.

In the present article, we propose a new method, *3D-EdgeAngle*, illustrating an objective way to measure the edge angle at virtual cross sections along an entire artefact edge in defined steps and measurements at different distances perpendicular to the edge. With this method, the edge angle can be measured at any point in a high resolution depending on the research question. The method is applicable to any 3D model, is script-based and semi-automated. It provides a reproducible approach, systematically error-avoiding and resulting in quantitative and statistically evaluable data.

## 2. Materials and methods

A detailed methodology (capturing and processing the 3D model, calculating the edge angle, statistical analysis) can be found on protocols.io (https://dx.doi.org/10.17504/protocols.io.81wgb6j51lpk/v1). Thus, the materials and methods described here should be seen as an overview and not as a replacement to the protocol.

### 2.1 Experimental, archaeological, and standard samples

**Fig 1:**
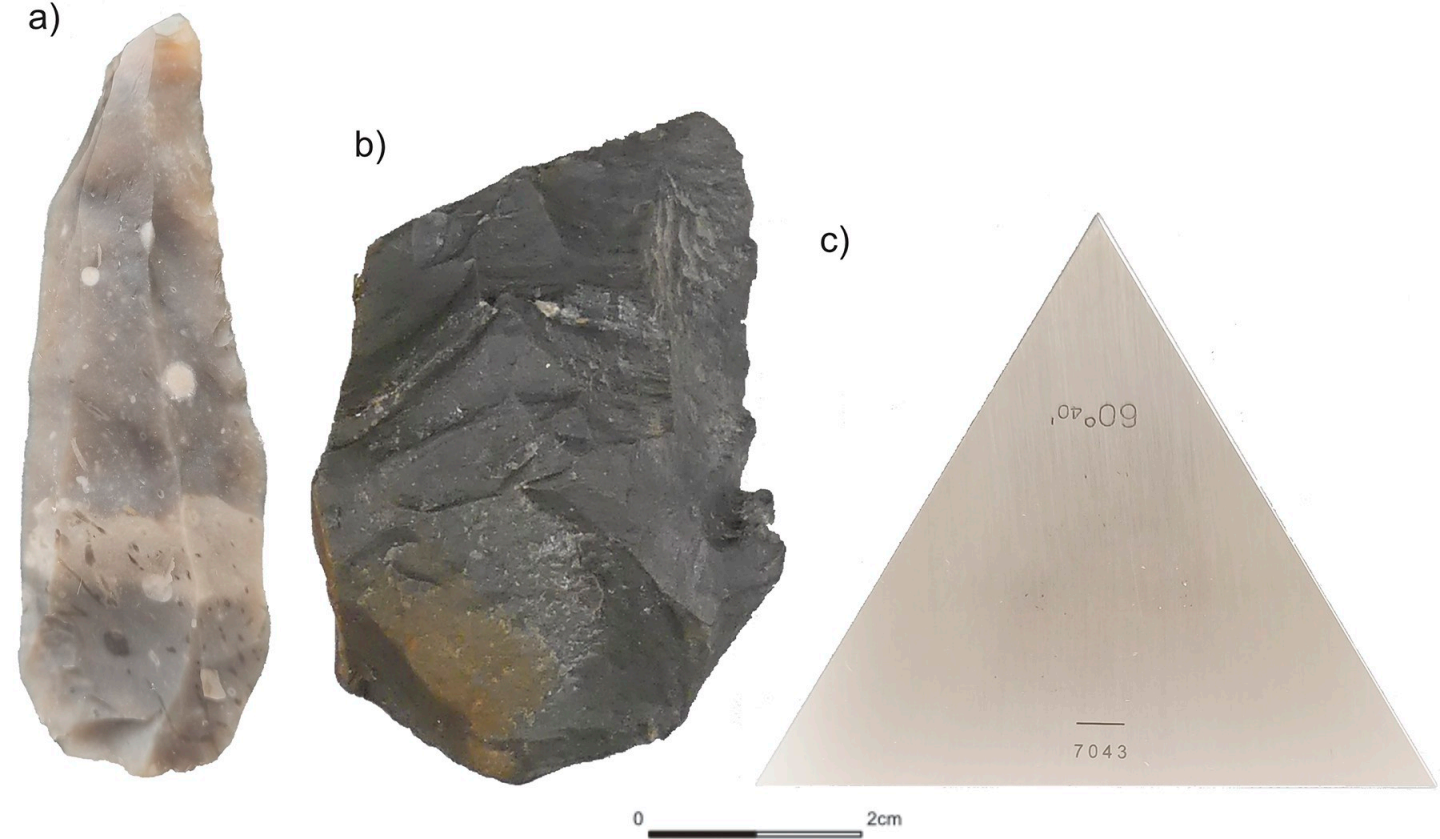
The three objects selected to demonstrate the applicability of *3D-EdgeAngle*: a) EAP-flake, b) BU-072 and c) WEM-60.

The application of the *3D-EdgeAngle* method is based on the use of a 3D model. To demonstrate the applicability of the method, we selected three different objects (**Fig 1**). The three objects comprise an experimental flake (1), an archaeological artefact (2), and a calibrated standard angle (gauge block; 3). The two lithic samples were selected due to their distinct edge modification, whereas the gauge block is taken as a reference and control sample, since it has, in contrast to the two other objects, a known edge angle.

(1) The first object (EAP-flake) is an experimental elongated laminar flake. In this case, the raw material is Baltic flint, and one edge of the flake is retouched in the distal part. The retouch is applied on the dorsal face and is performed marginally, i.e., minimally invasive.

(2) The second item (BU-072) is an archaeological artefact, a Keilmesser (bifacially backed knife) from the Late Middle Palaeolithic site Buhlen, Germany [30 for details about the artefact, 31]. The tool is made of black, shiny silicified schist and has one distinct active edge, shaped through bifacial flat and invasive retouch. The edge of the artefact, however, is largely characterised by the scar of a lateral tranchet blow applied to one of the tool’s surfaces.

(3) As a third item (WEM-60) we chose a calibrated standard angle (gauge block) made of steel. The gauge block has a certified nominal value of 60°40’ (~60.67°) and serves as a reference and control sample.

### 2.2 3D scanning

The three samples were scanned with an AICON smartSCAN-HE R8 from the manufacturer Hexagon, featuring a blue light LED and two black and white cameras with 8 megapixels each. The S-150 FOV (field of view) used has a point-to-point distance of 33 μm. Identical settings were used for all scans; scans were exported afterwards in an STL-format. The gauge block needed additional target marks on the surface due to its reflecting and homogeneous surface. One prerequisite for the application of 3D-EdegeAngle is a closed 3D model. The closed 3D models of the three items can be found on Zenodo (https://doi.org/10.5281/zenodo.7326241).

### 2.3 Digitalisation of the edge

With a closed 3D model as a basis, some preparatory steps are necessary before calculating the edge angle: the digitalisation of the edge. The edge of interest for calculating edge angles needs to be defined. Although the three samples have more than one edge, to explain the method, in this paper we only digitalised and used one edge per sample (retouched, active edge in the case of BU-072 and EAP-flake). In the case of a real analysis of archaeological tools, we would recommend defining a polyline for every tool edge to separate the functional interpretation from the analysis.

#### 2.3.1 ‘surface curve’

**Fig 2:**
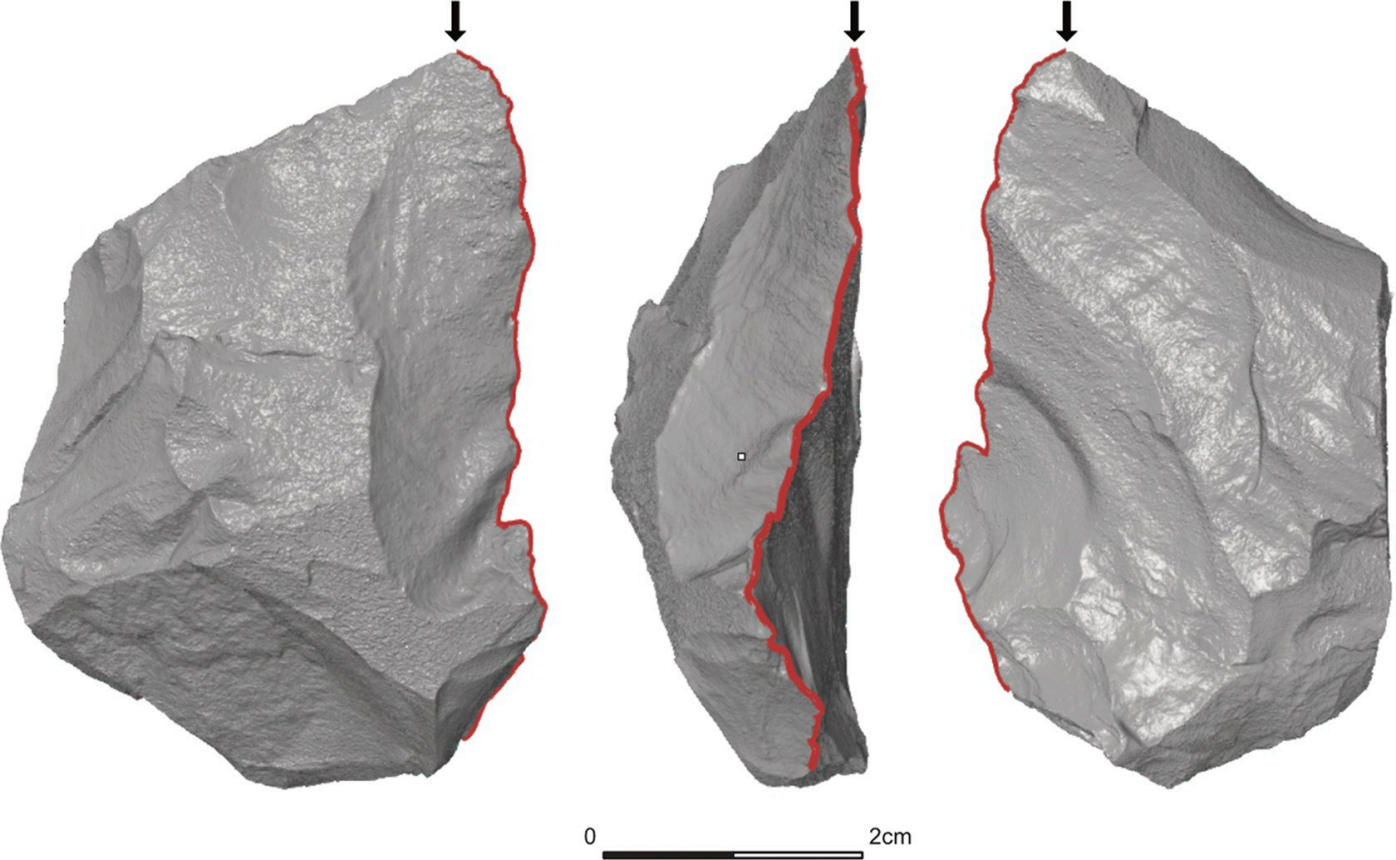
Upper (left), lateral (middle) and lower (right) surfaces of a Keilmesser from Buhlen (BU-072) extracted from a 3D scan. The red line indicates the ‘surface curve’ and follows the active edge of the tool. The errors indicate the beginning of the ‘surface curve’.

Since archaeological tools are variable in their morphology and display a complex geometry, this is a step that has to be done manually to reach the intended accuracy. Attempts to perform the digitalisation of the edge automatically have led to a loss of detail without reflecting the geometry of the edge in precision and accuracy. Thus, a ‘surface curve’ has been added manually to each 3D model, using the free version of GOM Inspect. This means, a polyline was defined by tracking the edge of the object as precisely as possible and later exported in an IGES-format (vertical black line on **Fig 2**).

#### 2.3.2 Reference curve and sections

The following steps were performed automatically with scripts using GOM Inspect Pro (licensed version). Alternatively, these steps can also be done manually using the free version GOM Inspect (see protocol on Zenodo: https://dx.doi.org/10.17504/protocols.io.81wgb6j51lpk/v1, step case: Manual version) and a programming language (e.g. Python or R). It has to be noted, however, that GOM Inspect is exclusively compatible with Windows operating systems. This also applies to the current release of GOM (now called ZEISS Inspect).

**Fig 3:**
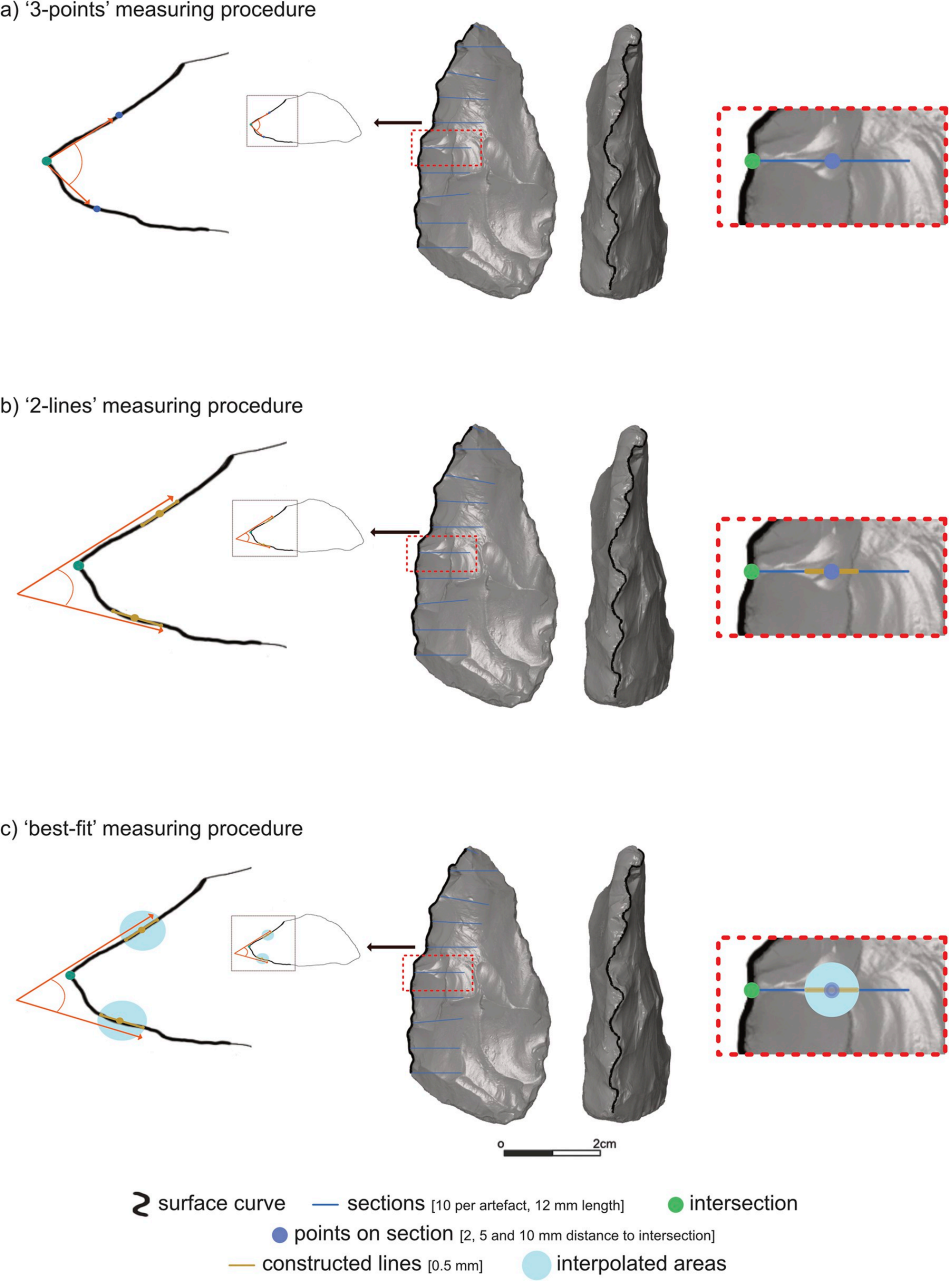
Schematic illustration of the *3D-EdgeAngle* method demonstrated on the 3D scan of a bifacial archaeological artefact. The scan displays the flat lower surface (middle left) and the active edge (middle right). The left part of the illustration shows the measurement procedures explained on the cross-section of the artefact at the position of the fifth section. The two blue points per procedure are placed on the section in a defined distance (blue points = points on section, golden lines = = constructed lines). The values mentioned in the legend are the parameters presented and used in the study.

The scripts were written in Python and were used with the Python Interface for GOM Inspect Pro (Zenodo: https://dx.doi.org/10.17504/protocols.io.81wgb6j51lpk/v1, step case: Script-based version). The software replaces the ‘surface curve’ polyline with a ‘reference curve’, which is slightly straightened by smoothing the changes in direction, reducing the point distance to 1 mm. This parameter is chosen based on various tries according to the resolution of the 3D models used here and therefore not a universal parameter. It can be assumed that a decreasing 3D model resolution requires a higher point distance. The ‘reference curve’ is used only for the purpose of defining the position of sections alongside the edge to be studied. The sections are polylines originating from the ‘reference curve’ and following the artefact surface perpendicularly and in a defined length (blue lines on **Fig 3**; each green point indicates the intersection between the vertical ‘surface curve’ and the section). In the case of using the ‘surface curve’ instead of the ‘reference curve’ polyline, the sections would not always be perpendicularly orientated because of the small changes in direction, which are the result of the detailed and complex artefact surface. After generating the ‘sections’, the ‘reference curve’ is not needed anymore. All further steps use the ‘surface curve’ created prior.

### 2.4 Measuring procedures

In this study, we introduce three possible measuring procedures to calculate edge angles (**Fig 3**). Also, these steps are performed automatically in GOM Inspect Pro by the use of a Python script. Parameters, which can be freely adjusted for the described measuring procedures, are explained below (see 2.4.4).

#### 2.4.1 ‘3-points’

The ‘3-points’ measuring procedure is modelled on the previously mentioned ‘*caliper method’* described by H. Dibble and M. Bernard in 1980 [20]. Whereas the result of the ‘caliper method’ is based on a measurement taken by a modified calliper at a known distance from the edge of the artefact by using a formula (edge angle: ($\theta =2\left[Tan^{-1}(\frac{.5T}{D})\right]);\;\theta$ = unknown edge angle, D = constant distance from the needle points to the edge of the vertical bar, T = thickness measurement), the ‘3-points’ digital measurement uses a more simple approach. Here, the same topographical indices represented by 3 points are used, but instead of a customised formula, the script applies the GOM Inspect built-in function to calculate the angle from 3 points (**Fig 3A**). The first point (green dot on **Fig 3A**) is the intersection between the vertical polyline (the ‘surface curve’), and each section. The two other points (blue dots on **Fig 3A**) are placed on the section, each on one surface, at a defined distance (cf. 2.4.4 Parameters) following the topography of the surface away from the intersection. Following this triangulation, the edge angle can be calculated between these three points (oranges lines on **Fig 3A**).

#### 2.4.2 ‘2-lines’

The second measuring procedure is the ‘2-lines’ measurement. This approach takes the intersection between the two polylines (‘surface curve’ and section(s)), as described in the first method, as one point (green dot on **Fig 3B**). Beginning with this point, it follows the section on both surfaces for a given distance (cf. 2.4.4 Parameters; blue dots on **Fig 3B**). Up until this step, the two procedures are identical. Unlike the previous procedure, these points on the section (blue dots on **Fig 3B**) are used as starting positions for the definition of two constructed lines (golden lines on **Fig 3**). The lines have a defined length and take the points as a centre from where they expand in both directions. The extremities of these lines are placed on the section (blue lines on **Fig 3B**). Thus, the calculation of the edge angle takes the two lines into account.

#### 2.4.3 ‘best-fit’

The third way of calculating the edge angle, the ‘best-fit’ procedure, is similar to the ‘2-lines’ measurement. The difference is that the lines are constructed by finding the best fit Using the built-in function ‘best-fit’ in GOM Inspect) along a defined length of the section. Therefore, the extremities of these lines are not necessarily placed on the section (blue lines on **Fig 3C**). This has the advantage of reducing a possible error caused by small changes in the section direction.

#### 2.4.4 Parameters

The parameters chosen for presenting the method are exemplary. In total, three different parameters are relevant for the proposed edge angle calculations. One of these three is the number of sections defined along the tool’s edge, chosen here to be ten sections per tool. It is possible to choose a fixed number of equidistant sections per tool, as done here, or to define steps, e.g., every few millimetres. Additionally, the length of the sections has to be defined, which are 12 mm long in this case.

Another parameter is the distance between the intersection (green dots on **Fig 3**) and the points on the sections (blue dots on **Fig 3**). In all three described measuring procedures, the intersection between the surface curve and the section determines one crucial point, but the other two points per measurement have to be defined numerically. Implemented values in this study are 2 mm, 5 mm, and 10 mm, but they can be varied freely. A script was written and used in R (programming language for statistical computing and graphics) to extract the measurements at these distances. The script can be found on Zenodo as open access (https://doi.org/10.5281/zenodo.7961582).

The last parameter is the lengths of the two constructed lines used in the ‘2-lines’ and ‘best- fit’ measuring procedures, which is here always 0.5 mm. Also, this length can be freely adjusted.

### 2.5 Statistical evaluation

The data analysis was performed in the open-source programming language Python. After loading and checking the data for consistency, a visual data analysis was performed as a first step. When several features of the data were pooled, e.g. the section or the distance to intersection, kernel density estimates were used to visualize the distributions of values, resulting in so-called violin plots [32,33].

In the second step, a quantitative comparison between the methods was conducted, requiring a statistical quality measure. Under the assumption that the first and second statistical moments of the quality measure are dominant, the maximum entropy principle [34 chapter 7.2.2 and references therein] yields the mean squared error as the suitable quality measure, which was consequently evaluated on the control sample. Scripts and the results are freely available on Zenodo (https://doi.org/10.5281/zenodo.7961582).

## 3. Results

The three different measurement procedures were applied to the three samples and the results compared. The results for the standard angle are illustrated by testing two models respectively: 1) the ten sections along the edge are pooled together to illustrate the edge angle results for the three distances and 2) the three distances (2 mm, 5 mm, 10 mm) along the sections are pooled together to show the edge angles for the ten sections. By illustrating the data in that way, edge angle changes along the edge and also from the intersection towards the surface, can be detected and highlighted, detailing accuracy and precision of the method and the measuring procedures.

### 3.1 Calibrated standard angle (gauge block)

**Fig 4:**
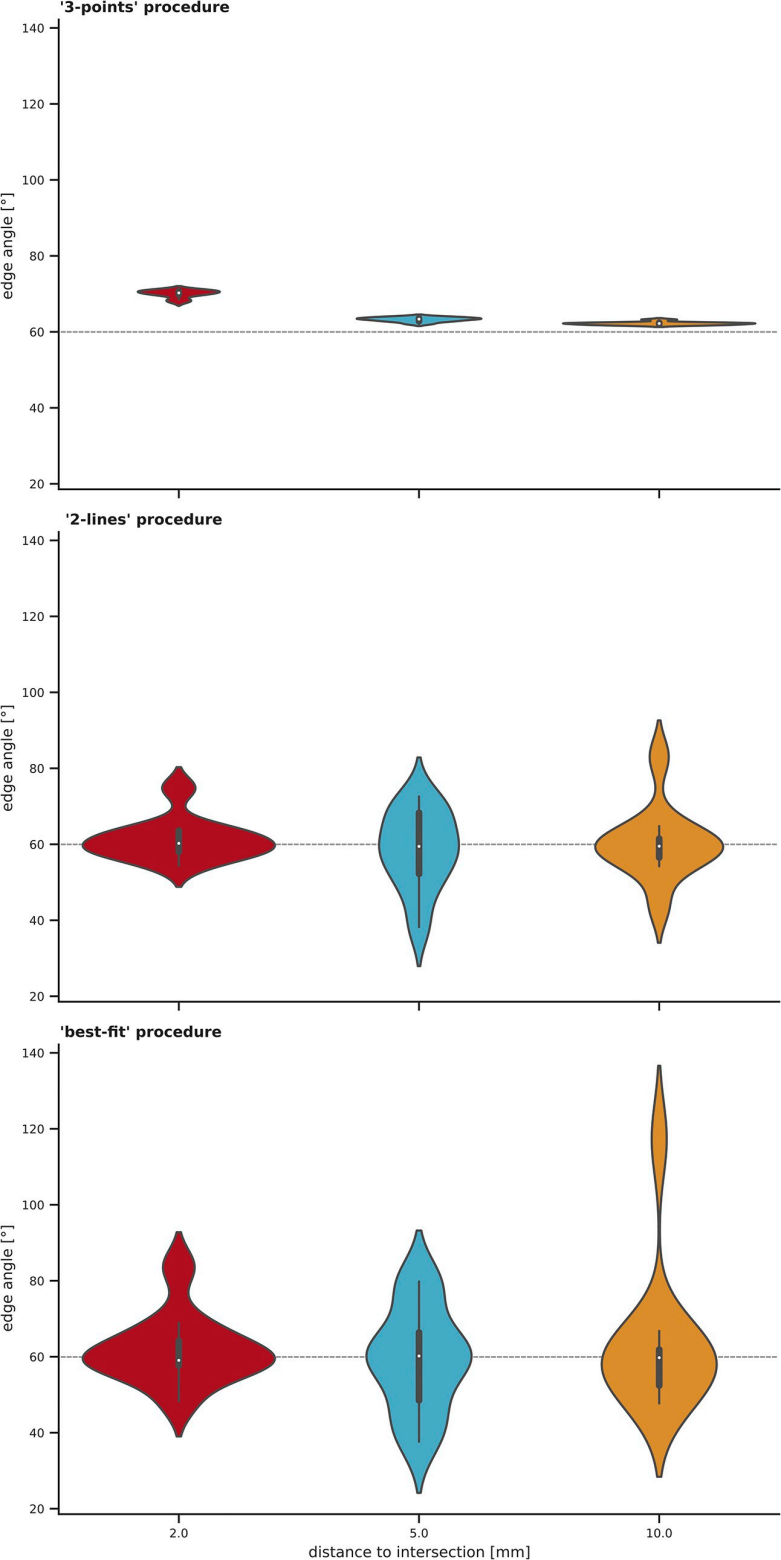
Edge angle measurements taken on the precision standard angle (WEM-60) at 2 mm (red plot), 5 mm (blue plot), and 10 mm (yellow plot) distance from the intersection. For each plot, the x axis represents the distance to intersection in mm, while the y axis shows the edge angle in degree. The ten sections along the edge (numbered from the distal (= 1) to proximal end (= 2) of the object) are pooled together to illustrate the edge angle results for the three different measuring procedures.

The validity of the method was assessed by introducing the calibrated standard angle (gauge block, WEM) as a control sample. The gauge block is calibrated precisely at 60°40’. For visualisation purposes, the graphic representation is plotted on a 60° baseline. Additionally, in the following, we refer to 60° although the true angle is a few arcseconds bigger. When pooling the ten sections (**Fig 4**), the data differs for the three procedures. The results calculated with the ‘3-points’ procedure vary from the nominal 60°, especially for the 2 mm distance. While the median for the ‘2-lines’ and ‘best-fit’ is 60°, the probability density of the data is spread further, meaning, the data differs and deviates from 60° through all the sections. By contrast, the ‘3-points’ measuring procedure shows more consistent, precise data and spreads less.

**Fig 5:**
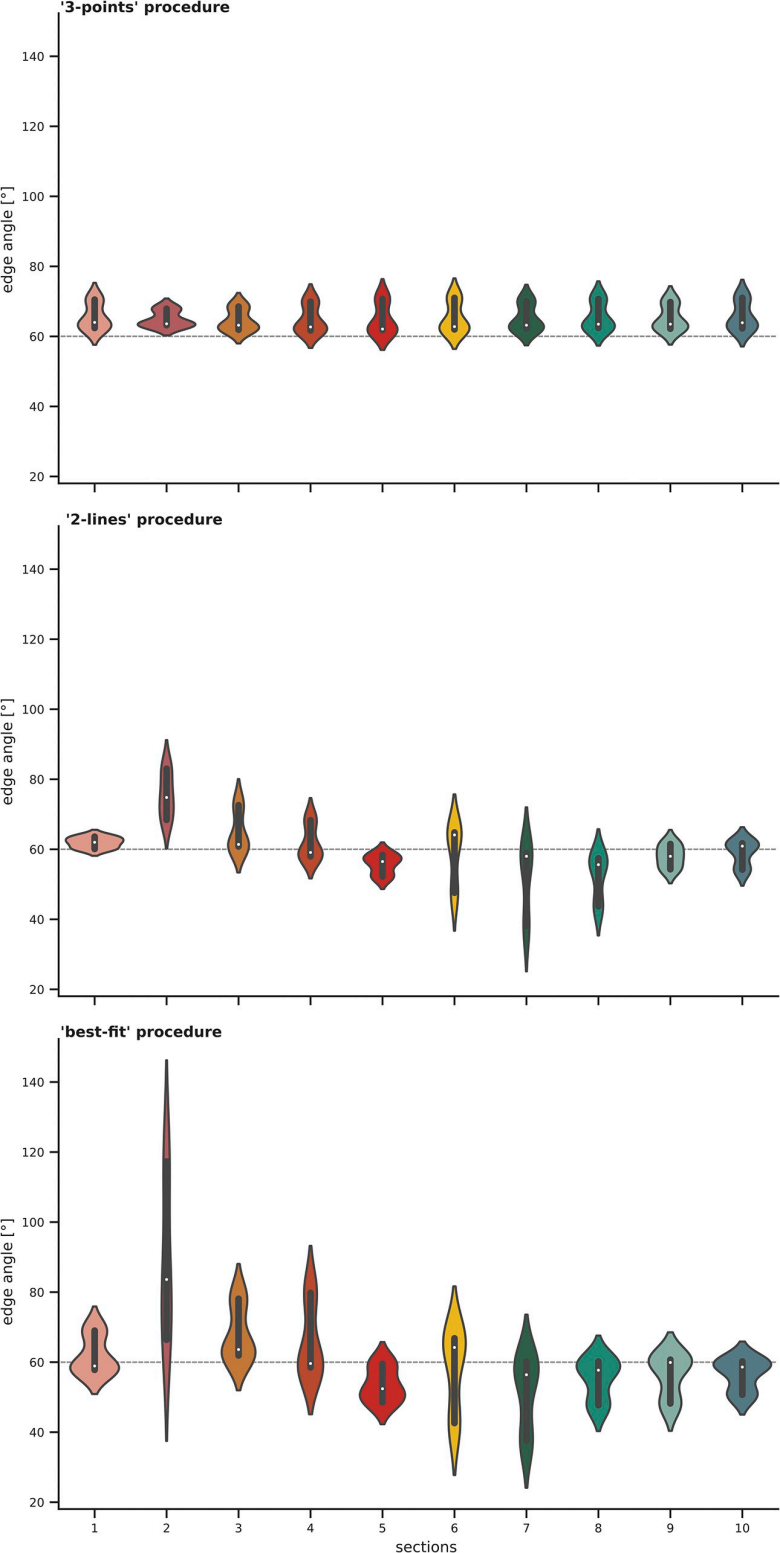
Edge angle measurements taken on the precision standard angle (WEM-60) at section one to ten. The three distances (2 mm, 5 mm, 10 mm) along the sections are pooled together to show the edge angle results for the three different measuring procedures. For each plot, the x axis represents the ten sections, while the y axis shows the edge angle in degree.

When instead pooling the distances, the results show similar trends (**Fig 5**).

Going further, the mean square error was evaluated as a statistical quality measure under the assumption that the true angle for all measurements is 60° (**Table 1**). The minimum squared error is realised by the ‘3-points’ measuring procedure at the 10.0 mm distance from the intersection.

**Table 1:** Results for the calculation of the mean squared error as a statistical quality measure for the standard angle (gauge block) under the assumption that the true angle for all sections is 60°.

measurement procedure	distance to intersection	mean squared error
**’3-points’**	2.0	100.99
	5.0	10.87
	10.0	5.05
**’2-lines’**	2.0	30.03
	5.0	101.62
	10.0	87.17
**’best-fit’**	2.0	83.05
	5.0	170.28
	10.0	363.01

Due to the incorporation of the micro-topography, the ‘2-lines’ and the ‘best-fit’ procedures of the *3D-EdgeAngle* method are highly sensitive. However, for the scale of analysis chosen here and for the resolution of the 3D model, the ‘best-fit’ procedure, which interpolates the lines within a defined distance, is too sensitive, producing potentially misleading results. For this reason, the results from the ‘best-fit’ will be excluded in the following, but will be addressed as reference values in the discussion.

### 3.2 Experimental flake and Keilmesser

**Fig 6:**
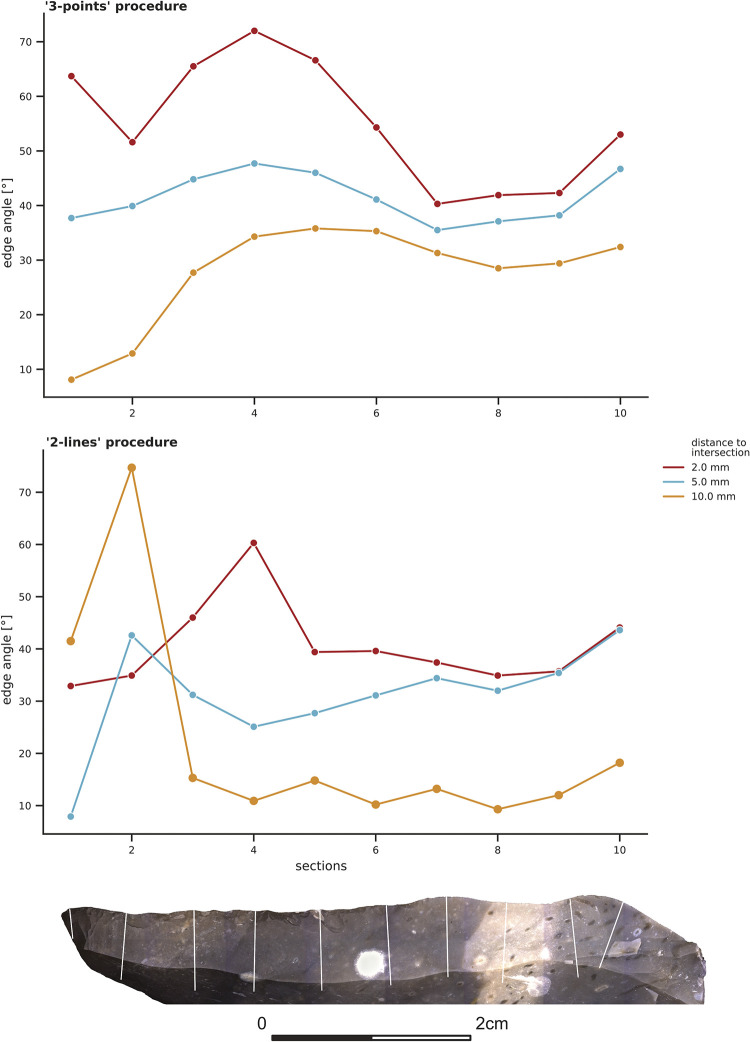
Edge angle measurements taken with the ‘3-points’ and ‘2-lines’ procedure on the experimental flake (EAP-flake) at section one to ten. The colours illustrate the results for the three distances (2 mm, 5 mm, 10 mm).

**Fig 7:**
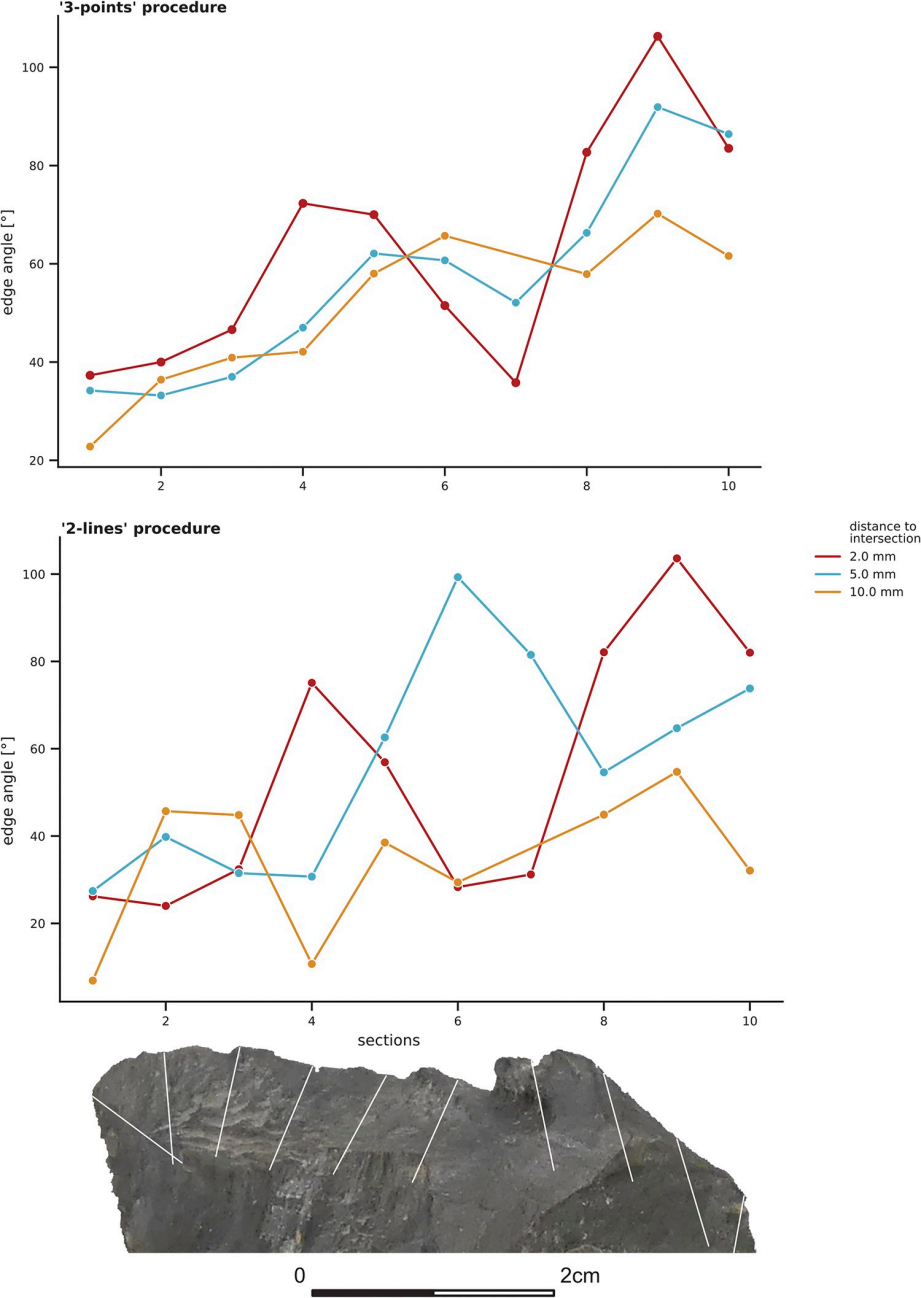
Edge angle measurements taken with the ‘3-points’ and ‘2-lines’ procedure on the Keilmesser (BU-072) at section one to ten. The colours illustrate the results for the three distances (2 mm, 5 mm, 10 mm). Photo taken by I. Görner.

The edge angle values for the two lithic samples (EAP-flake and BU-072; **Figs 6 and 7**) were calculated in the same way as the control sample before. Just as for the calibrated standard angle, the results of the ‘3-points’ measuring procedure differ from the results obtained by the ‘2-lines’ procedure. Moreover, the calculations at the various distances lead to distinctly different results. This is especially evident for the values obtained by the ‘2-lines’ procedure.

For the experimental flake (EAP-flake; **Fig 6**), the results of the ‘3-points’ procedure with the three distances to the intersection illustrated the same trend. Generally, the smaller the distance, the higher the edge angles. For the ‘2-lines’ procedure, the results obtained at the different distances to the intersection reflect the same overall trend but with higher variability, especially in the distal part (section 1 to 4).

The results for the Keilmesser (BU-072; **Fig 7**) calculated with the ‘3-points’ procedure are very similar for the 5 mm and 10 mm distance, whereas the values for the 2 mm distance deviate more. In contrast, the same sample yields diverse results for the various distances when edge angles are calculated with the ‘2-lines’ procedure.

## 4. Discussion

The edge of a stone tool, as part of the overall tool design, is a highly relevant variable when addressing tool function, performance, and use. While different (and mainly manual) methods exist to calculate the edge angle, they rarely result in systematic, quantitative, or reproducible data. *3D-EdgeAngle* presented here provides an algorithm-based method to measure the edge angles of 3D objects at cross sections along the entire tool edge in defined steps and at different distances perpendicular to the edge. *3D-EdgeAngle* includes three different procedures, which should be selected according to the object to be analysed and the scale of interest.

### 4.1 Influences of the parameter: Distance to intersection

**Fig 8:**
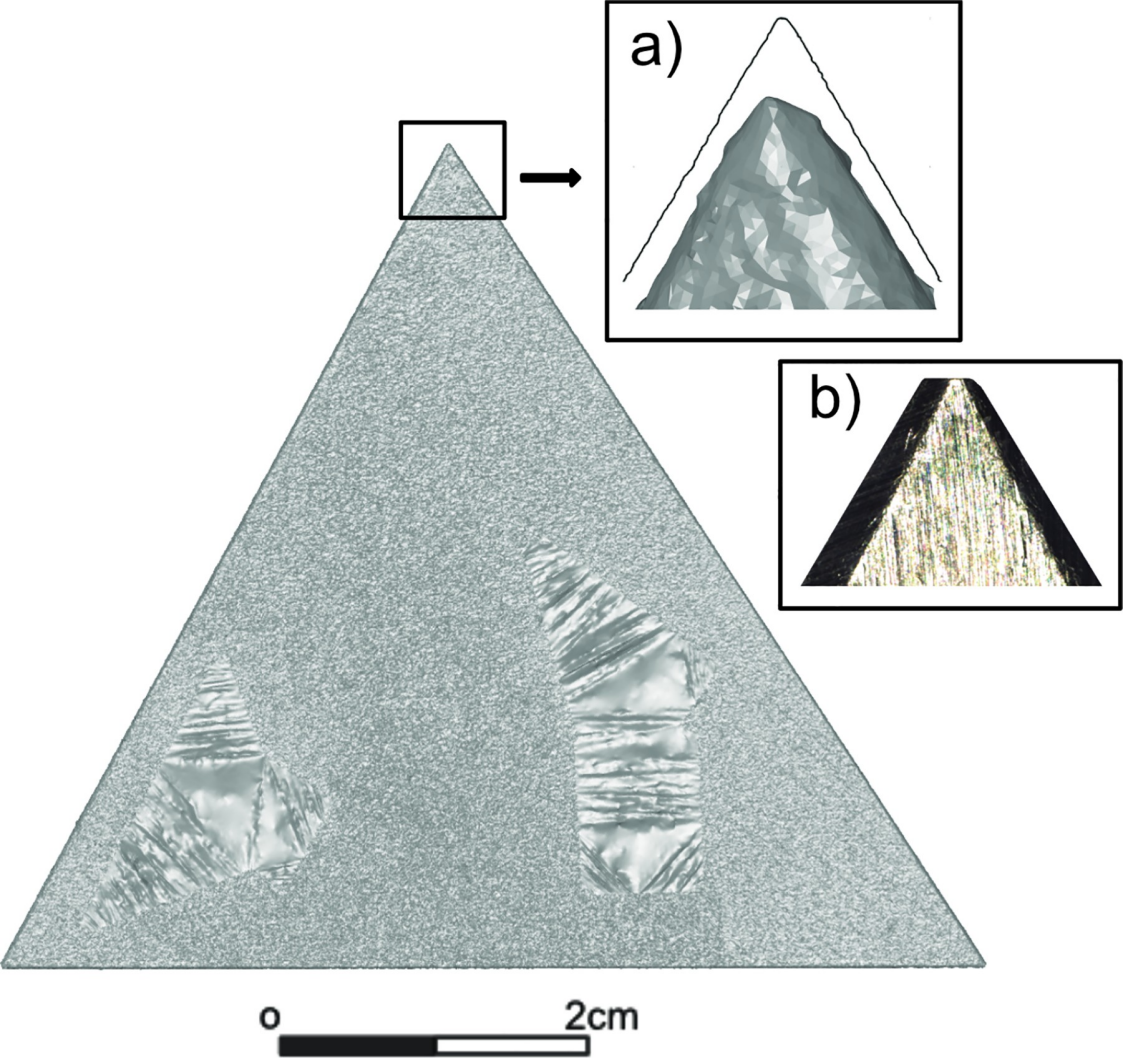
Image of the scanned calibrated standard angle (WEM-60). When zooming in (a), the meshes of the 3D model become visible and the sharp 60° angle appears dull/rounded. Imaging the standard angle with a digital microscope (ZEISS Smartzoom 5, 1.6x/0.10 objective, 140x total on-screen magnification), the EDF image (b) also confirms a rounded tip. The black line in the close-up (a) illustrates the first section. Note that the irregularities on the surface of the standard angle are the applied self-adhesive target marks.

The edge angles of the three studied objects could be reliably calculated with *3D-EdgeAngle*. The method is, however, only as good as the resolution and the quality of the 3D model used, which becomes apparent when checking the result for the calibrated standard angle calculated with the ‘3-points’ procedure at the 2 mm distance to intersection: the calculated edge angle is consistently different from the nominal 60° (**Fig 4**). Acute and sharp edges are often difficult to document with a 3D scanner. Zooming into the 3D model, the edges of the models appear blunt instead of sharp (**Fig 8A**), illustrating the inability to measure such fine features. As observed at high magnification, the calibrated standard angle is also not precisely 60°40’ at the very tip (**Fig 8B**). This may be due to regular production tolerances and the manufacturing processes of such steel standard angles, or it may be the results of physical damage at the tip. Therefore, the values calculated for the first millimetres are potentially higher than the values for the further distances to the intersection.

Although the method is predominantly algorithm- and script-based, the step of digitalising the edge by defining a polyline along the edge–the ‘surface curve’–still needs to be done manually. Defining the correct position of this polyline exactly at the intersection between two surfaces is challenging when the edge of the scanned artefact is curved (**Fig 2**). Positioning the polyline further towards one surface of the object will result in a higher than real edge angle value. To summarise, the measurements taken close to the edge are therefore likely to be more prone to errors, resulting in higher edge angle values at the first few distances to the intersection. Consequently, the chosen distance from the intersection can impact the result and depending on the artefact, research question and scale of interest, this parameter should be adjusted accordingly to avoid this region of inaccuracy very close to the tip.

### 4.2 Influences of the procedure

**Fig 9:**
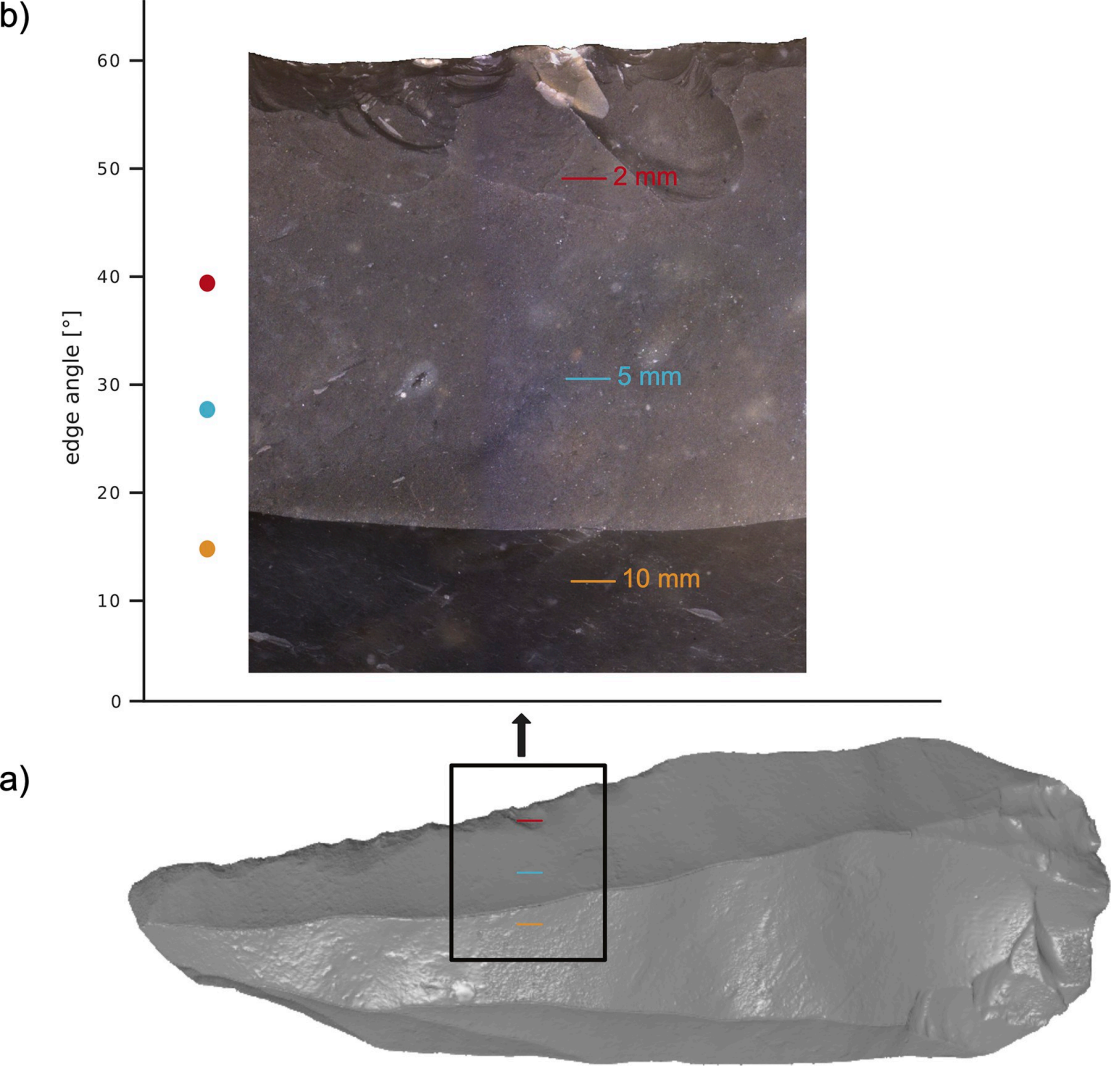
a) Edge angle measurements taken with the ‘2-lines’ procedure on the experimental flake (EAP-flake) at section five. b) The close-up demonstrates the morphological changes at the three distances (2 mm, 5 mm, 10 mm).

**Fig 10:**
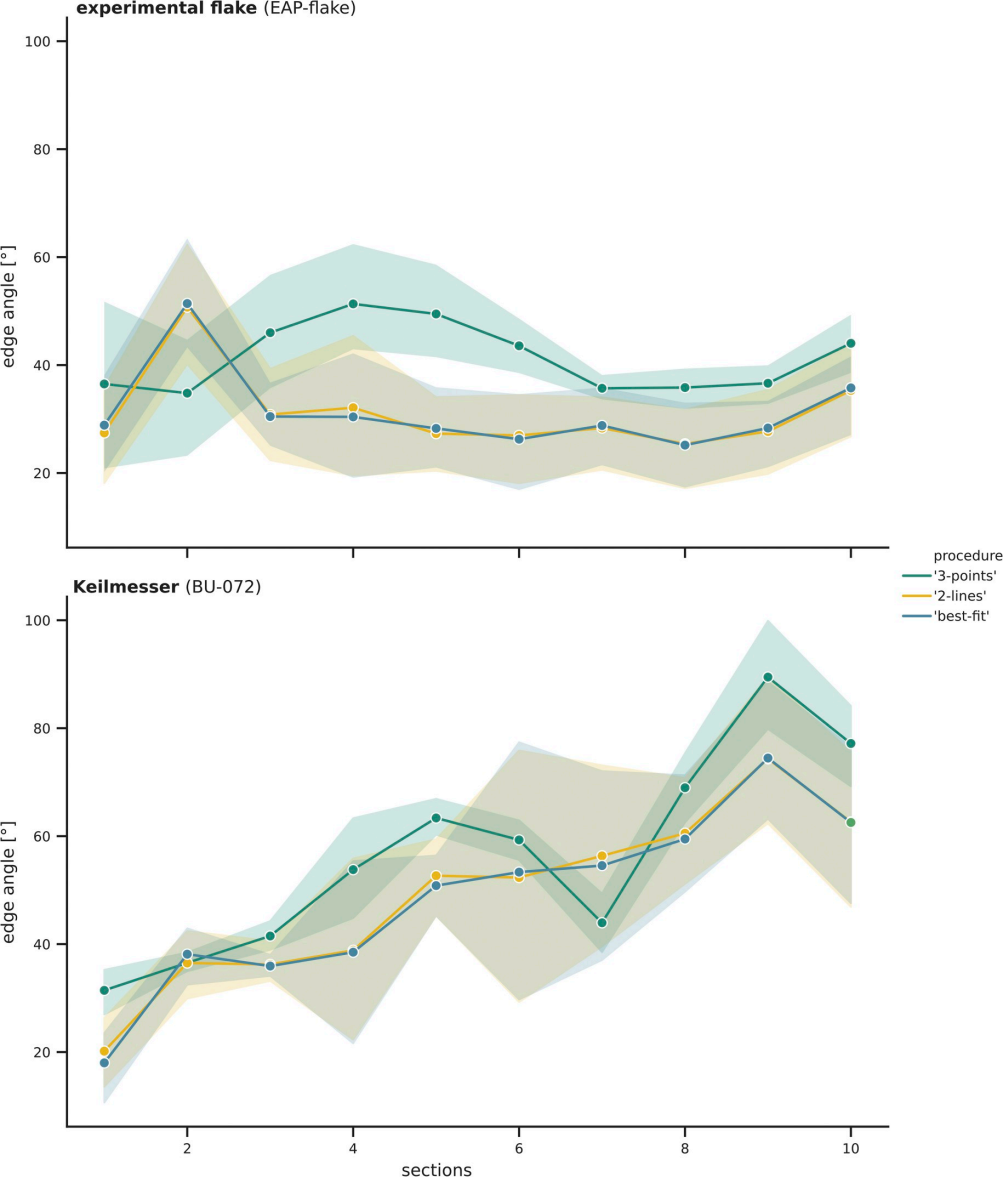
Edge angle measurements taken with the ‘3-points’, ‘2-lines’, and ‘best-fit’ procedures on the experimental flake (EAP-flake) and Keilmesser (BU-072) at section one to ten. The results of the measurements at the three distances to intersection (2 mm, 5 mm, 10 mm) are pooled together. The envelopes surrounding the data points outline the extremes of the curve.

In addition to the selected parameters, also the measurement procedure itself has an influence on the calculated values. Although the three measuring procedures lead to seemingly reliable results as they are close to 60° in the case of the gauge block (**Fig 4**), the ‘3-points’ procedure is closest to the actual value in the case of the gauge block. As described in the method section, this is reasoned by the way the calculation of the edge angle is done. In the case of the ‘3-points’ procedure, the calculation is based on a triangulation. Thus, we argue that the ‘3-points’ procedure is suitable for simple morphologies as represented by the calibrated standard angle. Due to the trigonometrical approach, the overall morphology of the scanned sample is taken into account for the calculation. Minor morphological changes such as a small, non-invasive retouch, inevitably get neglected. Based on the desired level of detail or scale of analysis, the ‘2-lines’ procedure might give a more realistic approximation of the edge angle. This can be illustrated with the results for the experimental flake. The values calculated with this procedure are rather dispersed and vary for the three different distances, since the morphology of the sample at 2 mm, 5 mm and 10 mm is not uniform. As highlighted in **Fig 9** (here section 5 as an example), the edge angle value taken at the 2 mm distance is still within the retouch on the dorsal face, whereas the sample is unmodified at the two other distances. The 10 mm distance even reaches across the ridge of one towards the second dorsal negative. Interestingly, the results are more dispersed within the sections one to five, which correlates with the retouch along the edge (**Fig 6**). At the sections six to ten, the flake is not retouched and edge angle values converge at 2 mm and 5 mm. The results obtained with the ‘3-points’ procedure follow similar trends, but the measuring procedure seems not sensitive enough to reflect certain surface modifications congruently in detail. This can also be seen when the results for the three distances are pooled together (**Fig 10**). While the ‘2-lines’ and the ‘best-fit’ procedures produced results much alike, the values calculated with the ‘3-points’ procedure differ up to 10° to 20°. Although they follow the same trend for the experimental flake at section six to ten (unretouched surface), within sections with retouch, the edge angle values vary. This is also visible for the Keilmesser, which has a more complex morphology due to retouch.

### 4.3 Accuracy and precision

The presented results draw attention to accuracy and precision as general challenges in edge angle measurements on lithic artefacts. The calculation of edge angles is prone to low precision, when measured manually or digitally. This was already pointed out by Dibble and Bernard [20], reporting a mean inter-observer variance for the ‘caliper method’ of 2.4° and for the ‘contact goniometer method’ of even 16.6°. Computer-based measuring methods reduce the inter-observer variance, but are not faultless either (e.g., virtual goniometer: 5°, [see 27]. Although the application of the *3D-EdgeAngle* method is script-based and semi-automated, it still prone to human error. Positioning of the polyline (‘surface curve’) along the edge is the only manually executed step but ultimately, all steps are transparent and/or reproducible.

Inclusion of the gauge block enabled an assessment of accuracies for the different measuring procedures. The highest level of accuracy could be achieved with the ‘3-points’ procedure for the calibrated standard angle.

### 4.4 Versatility of the method

Most measuring methods result in one average, overall edge angle value per artefact and may not yield sufficient data to address multiple research questions. *3D-EdgeAngle* offers significant potential in its versatility, as it allows for measurements at any point along the edge as well as perpendicular to the edge (distance to intersection). This type of data may shed light on questions about technological choices and the effect of retouch on the tool edges. For *3D-EdgeAngle*, precision can be influenced by either choosing the parameters (number of sections, distance to intersection and length of the lines) or by selecting the measurement procedure. We argue that the ‘3-points’ procedure is suitable for samples with simple morphologies (i.e., straight surfaces without drastic morphological changes), whereas more accuracy can be reached for complex morphologies with the ‘2-lines’ procedure. For studies with an increasingly detailed scale of analysis, also the ‘best-fit’ procedure might be an appropriate choice. The values calculated with the ‘2-lines’ as well as ‘best-fit’ procedures are mostly concurrent, but some exceptions could be noted. Based on the interpolation of points within a defined distance to the intersection, the results from the ‘best-fit’ procedure can also be misleading. For the scale selected here (for instance 0.5 mm for length for the constructed lines), this procedure is too error-prone. However, when adapting the parameters according to the scale of analysis, the procedure should be suitable for high-resolution analyses.

Due to the fact that accuracy and precision can be partially adjusted by the parameters and the measuring procedure chosen, *3D-EdgeAngle* can be used as a versatile method and applied to different artefact’ studies. A crucial example in the understanding of decision-making processes and the evolution of hominin technology is the study of stone core debitage and tool design. In this field, the analysis of features such as Exterior Platform Angle has been used as a key variable to understand hominin knapping behaviours (see [35] for a comprehensive synthesis on this topic). Accuracy in measuring angles is critical in these studies, and the *3D-EdgeAngle* has the potential to improve these measurements crucially.

## 5. Conclusion

With *3D-EdgeAngle*, we introduce a new digital tool to reliably calculate edge angles on any 3D object. Creating 3D models of archaeological artefacts is becoming a standard application in the discipline [36], allowing for analyses not possible otherwise. Simultaneously, the use of digital tools supports an increase in quantitative methods and automation, providing reproducible and statistically evaluable data. As demonstrated on a standard angle and two lithics, edge angles can be accurately and precisely measured at high resolution and semi-automatically. The edge angle of a tool, specifically of a lithic, is often associated with tool design, including technological variability, function, and use.

The possibility of measuring–as accurate and precisely as possible–the edge angles of lithic artefacts at any point along the edge and at different scales of analysis helps to better characterise functionality aspects of retouch activities and the relevance of variations along the edge, allowing archaeologists to recognise and interpret artefact variability in the archaeological record. *3D-EdgeAngle* is a versatile method and in its application not restricted to lithics, it can be used for all other 3D objects. In comparison to other methods, such as the ‘contact goniometer’ [20], extremely acute angles can be measured, too.

This way, significant improvements in the understanding of tool design can be gained, a necessary step towards the recognition of human technological adaptations and decision-making processes.
